# Neural Estimator of Information for Time-Series Data with Dependency

**DOI:** 10.3390/e23060641

**Published:** 2021-05-21

**Authors:** Sina Molavipour, Hamid Ghourchian, Germán Bassi, Mikael Skoglund

**Affiliations:** 1 School of Electrical Engineering and Computer Science (EECS), KTH Royal Institute of Technology, 100 44 Stockholm, Sweden; hamidgh@kth.se (H.G.); skoglund@kth.se (M.S.); 2 Ericsson Research, 164 83 Stockholm, Sweden; german.bassi@ericsson.com

**Keywords:** neural networks, conditional mutual information, directed information, Markov source, variational bound

## Abstract

Novel approaches to estimate information measures using neural networks are well-celebrated in recent years both in the information theory and machine learning communities. These neural-based estimators are shown to converge to the true values when estimating mutual information and conditional mutual information using independent samples. However, if the samples in the dataset are not independent, the consistency of these estimators requires further investigation. This is of particular interest for a more complex measure such as the directed information, which is pivotal in characterizing causality and is meaningful over time-dependent variables. The extension of the convergence proof for such cases is not trivial and demands further assumptions on the data. In this paper, we show that our neural estimator for conditional mutual information is consistent when the dataset is generated with samples of a stationary and ergodic source. In other words, we show that our information estimator using neural networks converges asymptotically to the true value with probability one. Besides universal functional approximation of neural networks, a core lemma to show the convergence is Birkhoff’s ergodic theorem. Additionally, we use the technique to estimate directed information and demonstrate the effectiveness of our approach in simulations.

## 1. Introduction

In recent decades, a tremendous effort has been done to explore capabilities of feed-forward networks and their application in various areas. Novel machine learning (ML) techniques go beyond conventional classification and regression tasks and enable revisiting well-known problems in fundamental areas such as information theory. The functional approximation power of neural networks is a compelling tool to be used for estimating information-theoretic quantities such as entropy, KL-divergence, mutual information (MI), and conditional mutual information (CMI). As an example, MI is estimated with neural networks in [1] where numerical results show notable improvements compared to the conventional methods for high-dimensional, correlated data.

Information-theoretic quantities are characterized by probability densities and most classical approaches aim at estimating the densities. These techniques may vary depending on whether the random variables are discrete or continuous. In this paper, we focus on continuous random variables. Examples of conventional non-parametric methods to estimate these quantities are histogram and partitioning techniques, where the densities are approximated and plugged-in into the definitions of the quantities, or methods based on the distance of the *k*-th nearest neighbor [2]. Despite vast applications of nearest neighbor methods for estimation of information-theoretic quantities, such as the proposed technique in [3], recent studies advocate using neural networks while simulations demonstrate that the accuracy of the estimations improves in several scenarios [1,4]. In particular, the results indicate that by increasing the dimension of the data, the bias of the estimation deteriorates less with neural estimators. In addition to superior performance, a neural estimator of information can be considered to be a stand-alone block and coupled in a larger network. The estimator can then be trained simultaneously with the rest of the network and measure the flow of information among variables of the network. Therefore, it facilitates the implementation of ML setups with constraints on information measures (e.g., information bottleneck [5] and representation learning [6]). These compelling features motivate exploring the benefits of neural networks to estimate other information measures and more complex data structures.

The cornerstone of neural estimators for MI is to approximate bounds on the relative entropy instead of computing it directly. These bounds are referred to as variational bounds and recently have gained attention due to their applications in ML problems. Examples are the lower bounds proposed originally in [7] by Donsker and Varadhan, and in [8] by Nguyen, Wainwright, and Jordan that are referred to as DV bound and NWJ bound, respectively. Several variants of these bounds have been reviewed in [9]. Variational bounds are tight, and the estimators proposed in [1,4,10,11] leverage this property and use neural networks to approximate the bounds and correspondingly the desired information measure. These estimators were shown to be consistent (i.e., the estimation converges asymptotically to the true value) and suitably estimate MI and CMI when the samples are independently and identically distributed (i.i.d.). However, in several applications such as time series analysis, natural language processing, or estimating information rates in communication channels with feedback, there exists a dependency among samples in the data. In this paper, we investigate analytically the convergence of our neural estimator and verify the performance of the method in estimating several information quantities.

Consider several random processes such that their realizations are dependent in time. In addition to common information-theoretic measures such as MI and CMI, more complex quantities can be studied that are paramount in representing these processes. For instance, the (temporal) causal relationship between two random processes has been expressed with quantities such as directed information (DI) [12,13] and transfer entropy (TE) [14]. Both DI and TE have a variety of applications in different areas. In communication systems, DI characterizes the capacity of a channel with feedback [15], while it has several other applications in venues including portfolio theory [16], source coding [17], and control theory [18] where DI is exploited as a measure of privacy in a cloud-based control setup. Additionally, DI was introduced as a measure of causal dependency in [19] which led to a series of works in that direction with applications in neuroscience [20,21] and social networks [22,23]. TE is also a well-celebrated measure in neuroscience [24,25], and the physics community [26,27] to quantify causality for time series. In this paper, we investigate capability of the neural estimator proposed in [11] to be used when the samples in the data are not generated independently.

Conventional approaches to estimate KL-divergence and MI such as nearest neighbor methods can be used for non-i.i.d. data; for example to estimate DI [28] and TE [29,30]. However, it is possible to leverage the benefits of neural estimators highlighted in [1] even though the data are generated from a source with dependency among its realizations. In a recent work [31], the authors estimate TE using the neural estimator for CMI introduced in [4]. Additionally, recurrent neural networks (RNN) are proposed in [32] to capture the time dependency to estimate DI. However, showing convergence of these estimators requires further theoretical investigation. Although the neural estimators are shown to be consistent in [1,4,11] for i.i.d. data, the extension of the proofs to dependent data needs to be addressed. In [32], the authors address the consistency of the estimation of DI by referring to universal approximation of RNN [33] and Breiman’s ergodic theorem [34]. Because RNNs are more complicated to be implemented and tuned, in this paper, we assume simple feed-forward neural networks, which were also proposed in [1,4,11] and in this paper. A conventional step to go beyond i.i.d. processes is to investigate stationary and ergodic Markov processes which have numerous applications in modeling real-world systems. Many convergence results for i.i.d. data such as the law of large numbers can be extended to ergodic processes; however, this generalization is not always trivial. The estimator proposed in [11] exhibits major improvements in estimating the CMI. Nevertheless, it is based on a *k*-nearest neighbors (*k*-NN) sampling technique which makes the extension of the convergence proofs to non-i.i.d. data more involved. The main contribution of this paper is to provide convergence results and consistency proofs for this neural estimator when the data are stationary and ergodic Markov.

The paper is organized as follows. Notations and basic definitions are introduced in Section 2. Then, in Section 3, the neural estimator and procedures are explained. Additionally, the convergence of the estimator is studied when the data are generated from a Markov source. Next, we provide simulation results in Section 4 for synthetic scenarios and verify the effectiveness of our technique in estimating CMI and DI. Finally, we conclude the paper in Section 5 and suggest potential future directions.

## 2. Preliminaries

We begin by describing the notation used throughout the paper, and the main definitions are explained afterwards. Then we review variational bounds which are the basis of our neural estimator.

### 2.1. Notation

Random variables and their realizations are denoted by capital and lower case letters, respectively. Given two integers *i* and *j*, a sequence of random variables $X_{i},X_{i+1},\ldots ,X_{j}$ is shown as $X_{i}^{j}$, or simply $X^{j}$ when i=1. For a stochastic processes Z, a randomly generated sample is denoted by random variable *Z*. We indicate sets with calligraphic notation (e.g., X). The space of *d*-dimensional real vectors is shown as $R^{d}$. The probability density function (PDF) of a random variable *X* at X=x is denoted by $p_{X}\left(x\right)$ or equivalently p(x), and the distribution of *X*, by $P_{X}$ or simply *P*. The PDF of multiple random variables $X_{1},\ldots ,X_{i}$ is $p_{X_{1}\;\ldots X_{i}}\left(x_{1},\ldots ,x_{i}\right)$ and for simplicity it is represented by $p(x_{1},\ldots ,x_{i})$ in the paper. For the distribution *P*, $E_{P}\left[\cdot \right]$ denotes the expectation with respect to its density p(·). All the logarithms are in base *e*.

The convergence of the sequence $X_{n}$ almost surely (or with probability one) to *X* is denoted by $X_{n}\overset{a.s.}{\to }X$ and is defined as:

$$P\;\left(\lim_{n\to \infty }X_{n}=X\right)=1.$$

### 2.2. Information Measures

The information-theoretic quantities of interest for this work can be written in terms of a KL-divergence, and the available neural estimators originally aim to estimate this quantity. For a random variable *X* with support $X\subseteq R^{d}$, the KL-divergence between two PDFs p(x) and q(x) is defined as:

(1) $$\begin{array}{c} D\left(p\left(x\right)\;∥\;q\left(x\right)\right):=E_{P}\left[\log\frac{p(X)}{q(X)}\right]. \end{array}$$

Then, CMI can be defined using KL-divergence as below:

(2) $$\begin{array}{c} I(X;Y|Z):=D(p(x,y,z)\;∥\;p(x|z)p(y,z)). \end{array}$$

where *Y* and *Z* are random variables with support on Y and Z, which are subsets of $R^{d}$. In this paper, we are focused on extending the estimators for CMI with non-i.i.d. data, where samples in time-series data might not be independently and identically distributed (e.g., generated from a Markov process); nonetheless, our method and consistency proofs are fairly general and can be applied for estimating KL-divergence as well. Consider a sequence of random samples ${\left\{\left(X_{i},Y_{i},Z_{i}\right)\right\}}_{i=1}^{n}$ generated from the joint process (X,Y,Z), where the samples are not necessarily i.i.d.. A simple step toward this extension is to verify that the previous neural estimators, e.g., [11], can be used to estimate I(X;Y|Z), where (X,Y,Z)∼p(x,y,z) and the processes (X,Y,Z) are Markov, as in the following assumption.

**Assumption** **1.** *(X,Y,Z) are jointly stationary and ergodic 1-st order Markov with marginal density p(x,y,z). The extension of the results to d-th order Markov is straightforward.*

**Figure 1:**
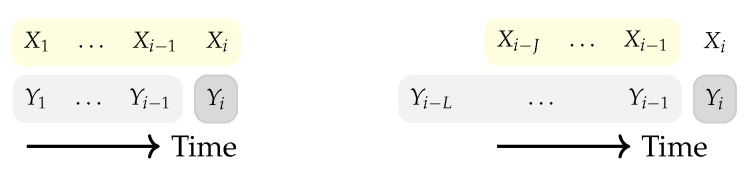
The memory considered for conditional mutual information terms in directed information (**left**) and transfer entropy (**right**) at time instance *i*. To compute directed information (**left**), the effect of $X^{i}$ (i.e., $X_{i}$ and all its past samples) on $Y_{i}$ is considered, while the history of $Y_{i}$ is excluded. However, for transfer entropy (**right**), the effect of $X_{i-J}^{i-1}$ (i.e., the previous *J* samples before $X_{i}$) on $Y_{i}$ is accounted for, while we exclude the history of $Y_{i}$. Note that the length of memories (*J* and *L*) for transfer entropy may differ.

To explore further in generalizing the neural estimators, it is possible to investigate their capability for information measures that rely on dependent random variables. Consider the pairs ${\left\{\left(X_{i},Y_{i}\right)\right\}}_{i=1}^{n}$ to be samples of the processes (X,Y). If the generated samples are dependent in time, it is possible to measure the causal relationship between the processes with quantities such as DI and TE, defined as below:

(3) $$\begin{array}{c} I(X^{n}\to Y^{n}):=\sum_{i=1}^{n}I\left(X^{i};Y_{i}|Y^{i-1}\right) \end{array}$$

(4) $$\begin{array}{c} T_{X\to Y}\left(i\right):=I(X_{i-J}^{i-1};Y_{i}|Y_{i-L}^{i-1}), \end{array}$$

where *J* and *L* are parameters of the TE that determine the length of memory to consider for **X** and Y, respectively. Both quantities are functions of the CMI and Figure 1 visualizes the corresponding variables in each CMI term for DI and TE. In particular, each CMI term in (3) quantifies the amount of shared information between $X^{i}$ and $Y_{i}$ conditioned on $Y^{i-1}$, i.e., it excludes the effect of the causal history of Y. In a general form, to express the causal effect of the process X on Y conditioning causally on Z, DI is normalized with respect to *n* which is defined below and denoted as directed information rate (DIR):

(5) $$\begin{array}{cc} I(X\to Y\;∥\;Z) & :=\lim_{n\to \infty }\frac{1}{n}I\left(X^{n}\to Y^{n}\;∥\;Z^{n}\right)\\ \; & =\lim_{n\to \infty }\frac{1}{n}\sum_{i=1}^{n}I\left(X^{i};Y_{i}|Y^{i-1},Z^{i}\right). \end{array}$$

By assuming the processes to be Markov, (5) can be simplified (see [23,35,36]). To be explicit, if both (X,Y,Z) and (Y,Z) are stationary and ergodic 1-st order Markov, from (5) the DIR can be simplified as:

(6) $$\begin{array}{c} I\left(X\to Y\;∥\;Z\right)=I\left(X^{2};Y_{2}|Y_{1},Z^{2}\right), \end{array}$$

where the CMI is with respect to the stationary density $p(x^{2},y^{2},z^{2})$ of the Markov model. To generalize this approach, let us define the *maximum Markov order* ($o_{\max}$) of a set of processes to be the minimum number *o* such that the Markov order of the joint random variables of any subset of the processes is less than or equal to *o*. So if $o_{\max}=l$ for (X,Y,Z), then from (5) we can simplify the DIR term as:

(7) $$\begin{array}{c} I\left(X\to Y\;∥\;Z\right)=I\left(X^{l+1};Y_{l+1}|Y^{l},Z^{l+1}\right). \end{array}$$

The following example shows how DIR can be computed for a linear data model, and emphasizes on the difference when DIR is conditioned causally on another process.

**Example** **1.** *Consider the following linear model where ${\left\{W_{i}\right\}}_{i=1}^{\infty }$, ${\left\{W_{i}^{\prime }\right\}}_{i=1}^{\infty }$, and ${\left\{W_{i}^{\prime \prime }\right\}}_{i=1}^{\infty }$ are uncorrelated white Gaussian noises with variances $\sigma_{x}^{2}$,$\sigma_{y}^{2}$, and $\sigma_{z}^{2}$ respectively:*

$$\begin{array}{c} \left\{\begin{array}{c} X_{i}=W_{i}\\ Y_{i}=a\;Y_{i-1}+Z_{i-1}+W_{i}^{\prime }\\ Z_{i}=X_{i}+W_{i}^{\prime \prime } \end{array}\right. \end{array}$$

*for some |a|<1, and $\left(X_{0},Y_{0},Z_{0}\right)$ are distributed according to the stationary distribution of the processes X, Y, and Z. This model holds in Assumption 1 and $o_{\max}=1$, so I(X→Y) can be computed as:*

$$\begin{array}{c} I\left(X\to Y\right)=I\left(X_{1}^{2};Y_{2}|Y_{1}\right)=\frac{1}{2}\log\left(1+\frac{\sigma_{x}^{2}}{\sigma_{y}^{2}+\sigma_{z}^{2}}\right), \end{array}$$

*while from (7):*

(8) $$\begin{array}{c} I\left(X\to Y\;∥\;Z\right)=I\left(X_{1}^{2};Y_{2}|Y_{1},Z_{1}^{2}\right)=0. \end{array}$$

As emphasized earlier, (7) holds when (X,Y,Z) and (Y,Z) are Markov with order *l*. Then the CMI estimators can be used potentially to estimate the DIR. However, the consistency of the estimation still needs to be investigated since the samples are not independent. Before introducing our technique, we review the basics for estimating information measures with neural networks.

### 2.3. Estimating the Variational Bound

The estimators proposed in [1,4,11] are all based on tight lower bounds on the KL-divergence, such as the *DV bound*, introduced in [7]:

(9) $$\begin{array}{c} D\left(p\left(x\right)\;∥\;q\left(x\right)\right)\geq \underset{f\in F}{\sup}E_{P}\left[f(X)\right]-\log E_{Q}\left[\exp(f(X))\right], \end{array}$$

where *p* and *q* are two PDFs defined over X with corresponding distributions *P* and *Q*, respectively, and F is any class of functions such that f:X→R, and the two expectations exist and are finite. Consider a neural network with parameters θ∈Θ, then F can be to the class of all functions constructed with this neural network by choosing different values for the parameters θ. In more details, let f(x) to be the end-to-end function of a neural network with parameters θ∈Θ and the optimization in the right hand side (RHS) of (9) is equivalent to optimizing over Θ (as performed in [1]). Nevertheless, we can leverage from the fact that the DV bound is tight when the function is chosen as:

(10) $$\begin{array}{c} f^{*}\left(x\right)=\log\frac{p(x)}{q(x)}\;\forall x\in X. \end{array}$$

Thus, the neural network can approximate $f^{*}\left(x\right)$ directly and the lower bound can be computed accordingly (as performed in [4,11]).

**Definition** **1.** *For the PDFs p(x,y,z) and p(x|z)p(y,z), define the corresponding distributions on X×Y×Z to be $\tilde{P}$ and $\tilde{Q}$, respectively.*

Since the CMI can be stated as a KL-divergence (2), the DV bound can be defined for CMI as bellow:

(11) $$\begin{array}{c} I\left(X;Y|Z\right)\geq \underset{f\in F}{\sup}E_{\tilde{P}}\left[f(X,Y,Z)\right]-\log E_{\tilde{Q}}\left[\exp(f(X,Y,Z))\right], \end{array}$$

and the bound is tight by choosing

(12) $$\begin{array}{c} f^{*}\left(x,y,z\right)=\log\frac{p(x,y,z)}{p(x|z)p(y,z)}\;\forall x,y,z\in X\times Y\times Z. \end{array}$$

The main barrier to compute this bound for $f^{*}\left(x,y,z\right)$ is that the densities are unknown. This challenge is addressed in [4,11] by proposing neural classifiers that can approximate $f^{*}\left(x,y,z\right)$ without knowing the densities. Below we review the steps of the estimation technique provided in [11]:

(1) Construct the joint batch, containing samples generated according to p(x,y,z).
(2) Construct the product batch, containing samples generated according to p(x|z)p(y,z).
(3) Train the neural network with a particular loss function, which we explain later, to approximate $f^{*}\left(x,y,z\right)$, i.e., the density ratio of $\frac{p(x,y,z)}{p(x|z)p(y,z)}$.
(4) Compute (11) using the batches and the approximated function.

To show the consistency of the estimation with this approach, it is crucial to verify if the empirical average with respect to each sample batch converges asymptotically to the corresponding expectations. Additionally, the neural network should be designed and trained to be capable of approximating the density ratio. For i.i.d. data samples, the authors in [4,11] provided the proofs in the form of concentration bounds. In this paper, we extend these proofs for non-i.i.d. data by providing convergence results for the special case of stationary and ergodic Markov processes. In the remainder of the paper, we denote the data by ${\left\{\left(X_{i},Y_{i},Z_{i}\right)\right\}}_{i=1}^{n}$ which are consecutive samples of the stationary Markov processes (X,Y,Z) with marginal PDF p(x,y,z).

## 3. Main Results

In this section, we describe our proposed neural estimator in detail. To create the batches, the estimator is equipped with a *k*-NN sampling block such that the empirical average over the samples converges to the expected mean. Next, we describe the roadmap to show the convergence of the estimation to the true value (i.e., consistency analysis).

### 3.1. Batch Construction

To create the joint batch it is sufficient to take $\left(X_{i},Y_{i},Z_{i}\right)$ randomly from the available data. Below we define the joint batch formally using an auxiliary random variable that indicates whether an instance is selected or not (see also Algorithm 1 for the implementation).
**Algorithm 1:** Construction of the joint batch
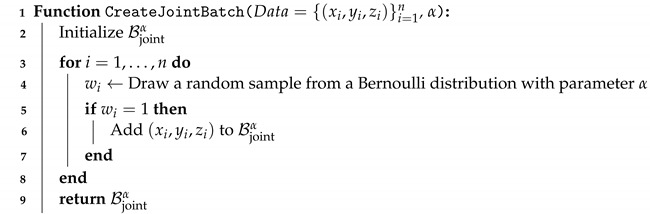

**Definition** **2** (Joint batch)**.** *Let $W_{i}∼Ber\left(\alpha \right)$ for i=1,…,n be independent random variables, and $I_{\alpha ,n}\left(W^{n}\right):=\left\{i|i\in \left\{1,\ldots ,n\right\},\;W_{i}=1\right\}$. Then $B_{joint}^{\alpha }$ is defined as*

(13) $$\begin{array}{c} B_{joint}^{\alpha }:=\left\{\left(X_{i},Y_{i},Z_{i}\right)|i\in I_{\alpha ,n}\right\}, \end{array}$$

*where we use $I_{\alpha ,n}$ to simplify the notation.*

Please note that by the law of large numbers, the length of the joint batch is asymptotically αn. Next, to construct the product batch we use the method based on the *k*-NN technique, which is introduced in [11]. Below we define our method denoted by *isolated k-NN* technique, and explain how the product batch is constructed (see also Algorithm 2).
**Algorithm 2:** Construction of the product batch
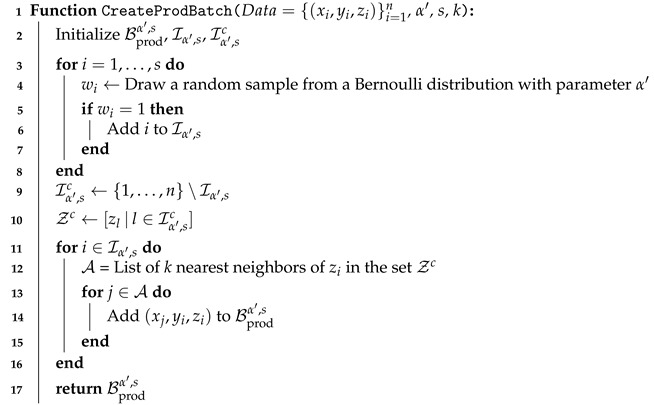

**Definition** **3** (Product batch)**.** *For s<n, let $W_{i}∼Bernoulli\left(\alpha^{\prime }\right)$ for i=1,…,s be independent random variables, and*

$$I_{\alpha^{\prime },s}\left(W^{s}\right):=\left\{i|i\in \left\{1,\ldots ,s\right\},\;W_{i}=1\right\}\;\&\;I_{\alpha^{\prime },s}^{c}\left(W^{s}\right):=\left\{1,\ldots ,n\right\}\backslash I_{\alpha^{\prime },s}\left(W^{s}\right).$$

*Then for any ζ∈Z and given the data ${\left\{\left(x_{i},y_{i},z_{i}\right)\right\}}_{i=1}^{n}$, define $A^{\alpha^{\prime },k,n,s}\left(\zeta ,z^{n},w^{s}\right)$ as the set of indices of the k nearest neighbors of ζ (by Euclidean distance) among $\left\{z_{i}\right\}$ for $i\in I_{\alpha^{\prime },s}^{c}\left(w^{s}\right)$. Formally, let $\pi :\left\{1,\ldots ,n-s\right\}\to I_{\alpha^{\prime },s}^{c}\left(W^{s}\right)$ be a bijection such that ${∥\zeta -z_{\pi (1)}∥}_{2}\leq \ldots \leq {∥\zeta -z_{\pi (n-s)}∥}_{2}$. Then, $A^{\alpha^{\prime },k,n,s}\left(\zeta ,z^{n},w^{s}\right):=\left\{\pi \left(1\right),\ldots ,\pi \left(k\right)\right\}$. So the product batch can be defined as:*

(14) $$\begin{array}{c} B_{prod}^{\alpha^{\prime },s}:=\left\{\left(X_{j(i)},Y_{i},Z_{i}\right)|i\in I_{\alpha^{\prime },s}\left(W^{s}\right),\;j\left(i\right)\in A^{\alpha^{\prime },k,n,s}\left(Z_{i},Z^{n},W^{s}\right)\right\}. \end{array}$$

**Figure 2:**
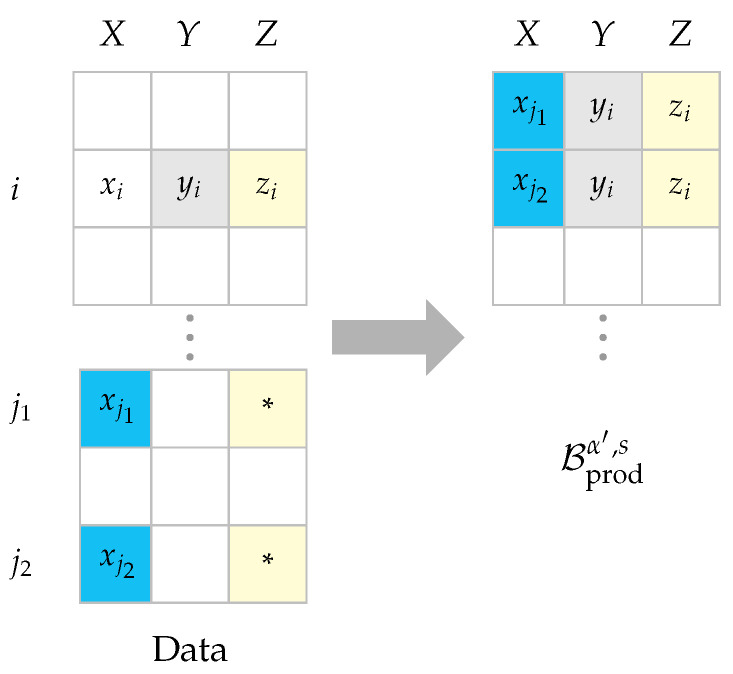
Construction of the product batch from the data set which is expressed as the left table. Let $w_{i}=1$, and the *z* component of the rows denoted with ‘*’ (indexed with $j_{1}$ and $j_{2}$) are in the *k* nearest neighborhood of $z_{i}$ for k=2. So we pack the triples $\left(x_{j_{1}},y_{i},z_{i}\right)$ and $\left(x_{j_{2}},y_{i},z_{i}\right)$ in the product batch as in the right table.

*Hereafter we use $I_{\alpha^{\prime },s}$, $I_{\alpha^{\prime },s}^{c}$, and $A^{\alpha^{\prime }}\left(\zeta \right)$ instead as the remaining parameters can be understood from the context. We refer to this sampling technique as* isolated *k*-NN *in the sequel. An example is also provided in Figure 2 for the case of k=2.*

**Remark** **1.** *Here we emphasize that the isolated indices are selected from the first s indices of samples while the neighbors can be searched among all n indices of data except the ones in $I_{\alpha^{\prime },s}\left(w^{s}\right)$. Additionally, note that the length of the product batch is $\alpha^{\prime }sk$ asymptotically as n→∞ because sk also tends to ∞ as we see later in the assumptions of Proposition 2.*

### 3.2. Training the Classifier

As explained earlier, the optimal function for a tight lower bound on the CMI is obtained by the density ratio and to compute that we use the functional approximation power of neural networks. Consider a feedforward neural network with the last layer equipped with the sigmoid function. The network is parameterized with $\theta \in \Theta \subseteq R^{h}$ where *h* is the number of parameters, and the neural network function is denoted by $\omega_{\theta }:X\times Y\times Z\to \left[0,1\right]$. For an input (X,Y,Z) of the network, let C∈{0,1} denote the class of the input which determines that the tuple is generated according to p(x,y,z) or p(x|z)p(y,z). To be explicit, the input is either picked from the joint batch (class C=1) or the product batch (class C=0), and the goal is to learn the network parameters such that it can distinguish the class of new (unseen) queries. Let the loss function be the binary cross-entropy function. So for ω to be any function with inputs (x,y,z) and ranging between [0,1], the expected loss is defined as:

(15) $$\begin{array}{c} L\left(\omega \right):=-E\left[C\log\omega (X,Y,Z)+(1-C)\log(1-\omega (X,Y,Z))\right]. \end{array}$$

It is well-established that by minimizing L(ω), the solution $\omega^{*}$ would represent the probability of classifying the input in the class C=1 given the input data, i.e., $P\;\left(C=1|x,y,z\right)$. In fact, as shown in [11] (Lemma 1) if the prior distribution on the classes is unbiased, by taking the derivative in (15) we have:

(16) $$\begin{array}{c} \Gamma \left(x,y,z\right)=\frac{p(x,y,z)}{p(x|z)p(y,z)}=\frac{\omega^{*}\left(x,y,z\right)}{1-\omega^{*}\left(x,y,z\right)}. \end{array}$$

So from (12) the optimal function can be expressed with Γ(x,y,z) as:

(17) $$\begin{array}{c} f^{*}\left(x,y,z\right)=\log\Gamma \left(x,y,z\right)\;\forall x,y,z\in X\times Y\times Z. \end{array}$$

Therefore, by training the neural network, we can approximate the optimal function $f^{*}\left(x,y,z\right)$ and estimate the lower bound for CMI.

Consider the neural network $\omega_{\theta }$, then the empirical loss function is defined as:

(18) $$\begin{array}{cc} \; & \;L_{emp}\left(\omega_{\theta }\right):=-\frac{1}{2\;|B_{joint}^{\alpha }|}\sum_{\left(X,Y,Z\right)\in B_{joint}^{\alpha }}\log\omega_{\theta }\left(X,Y,Z\right)\\ \; & \;-\frac{1}{2\;|B_{prod}^{\alpha^{\prime },s}|}\sum_{\left(X,Y,Z\right)\in B_{prod}^{\alpha^{\prime },s}}\log\left(1-\omega_{\theta }\left(X,Y,Z\right)\right), \end{array}$$

and the optimal parameters are obtained by solving the following problem:

(19) $$\begin{array}{c} \hat{\theta }:=\arg\min_{\theta }L_{emp}\left(\omega_{\theta }\right). \end{array}$$

Consequently, we can approximate the density ratio Γ(x,y,z) from (16):

(20) $$\begin{array}{c} \hat{\Gamma }\left(x,y,z\right)=\frac{\omega_{\hat{\theta }}\left(x,y,z\right)}{1-\omega_{\hat{\theta }}\left(x,y,z\right)}. \end{array}$$

To avoid having boundary values (i.e., $\omega_{\hat{\theta }}\left(x,y,z\right)$ close to zero or 1), the output of the neural network is clipped between [τ,1−τ] for some small τ>0.

**Remark** **2.** *Please note that $\hat{\Gamma }\left(x,y,z\right)$ approximates the density ratio, if the batch sizes $|B_{joint}^{\alpha }|$ and $|B_{prod}^{\alpha^{\prime },s}|$ are balanced. Otherwise, *(20)* requires a correction coefficient (see [11]). To fulfill this, given the number of samples n, one can choose the parameters such that $\alpha n=\alpha^{\prime }sk$. Then, by the law of large numbers, the batches will asymptotically be balanced.*

### 3.3. Estimation of the DV Bound

The final step in the estimation of CMI is to compute the lower bound (11) empirically using $\hat{\Gamma }\left(x,y,z\right)$. So by substituting the expectations with empirical averages with respect to samples in the joint and the product batch, the CMI estimator is defined as:

(21) $$\begin{array}{c} {\hat{I}}_{DV}^{n}\left(X;Y|Z\right):=\frac{1}{|B_{joint}^{\alpha }|}\sum_{\left(x,y,z\right)\in B_{joint}^{\alpha }}\log\hat{\Gamma }\left(x,y,z\right)+\log\frac{1}{|B_{prod}^{\alpha^{\prime },s}|}\sum_{\left(x,y,z\right)\in B_{prod}^{\alpha^{\prime },s}}\hat{\Gamma }\left(x,y,z\right). \end{array}$$

In practice, to mitigate the induced inaccuracy due to sampling from the original data, the training and estimation is repeated for several sampling trials. The steps for implementing the estimator are described in Algorithm 3. In the next part, we provide the convergence results for our estimator to validate substitution of the expectations in (11) with empirical averages with respect to the joint and the product batch. Then we show the convergence of the overall estimation to the true CMI value.
**Algorithm 3:** Estimation of CMI
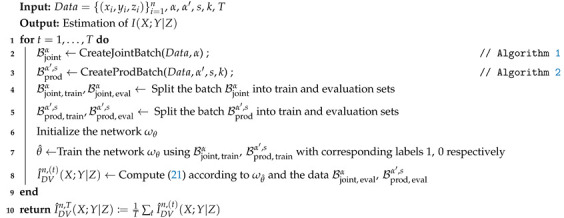


### 3.4. Consistency Analysis

The consistency of our neural estimator (i.e., showing that the estimator converges to its true value) is based on the universal functional approximation power of neural networks and concentration results for the samples collected in the joint batch and in the product batch using the *isolated k-NN*. Informally, Hornik’s functional approximation theorem [37] guarantees that feedforward neural networks are capable of fitting any continuous function. So depending on the true density of the data, there exists a choice of parameters $\tilde{\theta }$ that enables approximating the desired function with any arbitrary accuracy. Next, we show that the empirical loss function $L_{emp}\left(\omega_{\theta }\right)$ is concentrated around its mean $L(\omega_{\theta })$ for any θ. Combining these tools, we are able to minimize the empirical loss function as in (19) and we expect $\hat{\theta }$ to be close to $\tilde{\theta }$ asymptotically; thus, eventually $\hat{\Gamma }\left(x,y,z\right)$ properly approximates Γ(x,y,z). Additionally, the empirical computation of the DV bound is concentrated around the expected value which concludes the consistency of the end-to-end estimation of the CMI.

In this paper, we put the main focus on extending the concentration results provided in [11] (Proposition 1) with Markov assumption on data. Although conventionally many asymptotic results for i.i.d. data are assumed to hold for Markov data as well, the required extensions here are more involved due to the additional complexity of the *k*-NN method. In the following, we first show the convergence of the empirical average for the joint batch,

$${|B_{joint}^{\alpha }|}^{-1}\sum_{\left(X,Y,Z\right)\in B_{joint}^{\alpha }}g\left(X,Y,Z\right)\to E_{\tilde{P}}\left[g\left(X,Y,Z\right)\right],$$

where g(·) is any measurable function such that the expectation exists and is finite. As the product batch collects samples corresponding to the *k* nearest neighbors, convergence results for nearest neighbor regression are invoked to show that the empirical average for the product batch converges to the expectation with respect to the product distribution $\tilde{Q}$,

$${|B_{prod}^{\alpha^{\prime },s}|}^{-1}\sum_{\left(X,Y,Z\right)\in B_{prod}^{\alpha^{\prime },s}}g\left(X,Y,Z\right)\to E_{\tilde{Q}}\left[g\left(X,Y,Z\right)\right].$$

Then, we conclude the consistency of the overall estimation.

#### 3.4.1. Convergence for the Joint Batch

One well-known extension to the law of large numbers for non-i.i.d. processes is Birkhoff’s ergodic theorem, and is the basis of our proof to show the following proposition on the convergence of the sample average over the joint batch.

**Proposition** **1.** *Consider the sequence of random variables ${\left\{\left(X_{i},Y_{i},Z_{i}\right)\right\}}_{i=1}^{n}$ generated under Assumption 1. Consider the distribution $\tilde{P}$ in Definition 1, for any measurable function g(·) such that $E_{\tilde{P}}\left[g(X,Y,Z)\right]$ exists and is finite,*

(22) $$\begin{array}{c} \frac{1}{|B_{joint}^{\alpha }|}\sum_{\left(X,Y,Z\right)\in B_{joint}^{\alpha }}g\left(X,Y,Z\right)\overset{a.s.}{\to }E_{\tilde{P}}\left[g(X,Y,Z)\right]. \end{array}$$

**Proof.**  See Appendix A. ☐

#### 3.4.2. Convergence for the Product Batch

From Definition 3, the empirical summation over all samples in the product batch is equivalent to averaging $|I_{\alpha^{\prime },s}|$ times *k*-NN regressions. Considering a sequence of pairs ${\left\{\left(U_{i},V_{i}\right)\right\}}_{i=1}^{n}$ generated from stationary ergodic processes (U,V), the *k*-NN regression denotes the problem of estimating m(u):=E[V|U=u] with $m_{n}\left(u\right):=\frac{1}{k(n)}\sum_{j=1}^{k(n)}V_{r_{j}}$ where $r_{j}$ refers to the *j*-th nearest neighbor of *u* among $U_{1},\ldots ,U_{n}$. This problem has been well studied when the pairs $\left(U_{i},V_{i}\right)$ are generated i.i.d.. For example in [38], the authors show the convergence of $m_{n}\left(u\right)$ as:

(23) $$\begin{array}{c} P\;\left(\int |m_{n}\left(u\right)-m\left(u\right)|p\left(u\right)du\geq \epsilon \right)\leq \exp\left(-n\;a\;\epsilon^{2}\right), \end{array}$$

for some positive constant *a*, when k(n)→∞ and $\frac{k(n)}{n}\to 0$. However, if the pairs are not independent, convergence results require a more advanced condition denoted geometric ϕ-mixing condition or geometric ergodicity condition [39,40]. As argued in [39], the geometric ergodicity is not a restrictive statement and holds for a wide range of processes (see also [41]). For instance, linear autoregressive processes are geometrically ergodic [41] (Ch. 15.5.2). Below we review the ϕ-mixing condition.

**Definition** **4** (ϕ-mixing condition)**.** *A process U is ϕ-mixing if for a sequence ${\left\{\phi_{n}\right\}}_{n\in N}$ of positive numbers satisfying $\phi_{n}\to 0$ as n→∞, for any integer i>0 we have:*

(24) $$\begin{array}{c} \left|P(A\cap B)-P(A)P(B)\right|\leq \phi_{i}P\left(A\right), \end{array}$$

*for all n>0 and all sets A and B which are members of $\sigma (U_{1},\ldots ,U_{n})$ and $\sigma (U_{n+i},U_{n+i+1},\ldots )$, respectively. If $\left\{\phi_{n}\right\}$ is a geometric sequence, U is called geometrically ϕ-mixing.*

To show the convergence of the empirical average over the product batch, we make the following assumptions.

**Assumption** **2.** *The sequence ${\left\{\left(X_{i},Y_{i},Z_{i}\right)\right\}}_{i=1}^{n}$ is geometrically ϕ-mixing.*

**Assumption** **3.** *We assume that Y and Z are compact.*

**Proposition** **2.** *Let the sequence of random variables ${\left\{\left(X_{i},Y_{i},Z_{i}\right)\right\}}_{i=1}^{n}$ be generated under Assumptions 1–3, and we choose k(n) and s(n) such that:*

(25) $$\begin{array}{cc} \; & s(n)k(n)=n\\ \; & k(n)\to \infty \\ \; & s(n)\to \infty \\ \; & k\left(n\right)/{\left(\log n\right)}^{2}\to \infty . \end{array}$$

*Consider $\tilde{Q}$ defined in Definition 1. Then, for any function g(·) such that $E_{\tilde{Q}}\left[g(X,Y,Z)\right]$ exists and is finite, and additionally,*

(26) $$\begin{array}{c} |g\left(x,y_{1},z\right)-g\left(x,y_{2},z\right)|<L_{g}\left|y_{1}-y_{2}\right|\;\forall x\in X,\;z\in Z,\;y_{1},y_{2}\in Y, \end{array}$$

*where $L_{g}>0$ is the Lipschitz constant, we have that:*

(27) $$\begin{array}{c} \frac{1}{|B_{prod}^{\alpha^{\prime },s}|}\sum_{\left(X,Y,Z\right)\in B_{prod}^{\alpha^{\prime },s}}g\left(X,Y,Z\right)\overset{a.s.}{\to }E_{\tilde{Q}}\left[g(X,Y,Z)\right]. \end{array}$$

**Proof.**  See Appendix B. ☐

**Remark** **3.** *Examples of choices for k(n) and s(n) satisfying *(25)* are for instance $k\left(n\right)=n^{\frac{1}{2}}$ and $k\left(n\right)={\left(\log n\right)}^{2+\epsilon }$ for some ϵ>0. Please note that in [11], the consistencies are shown when $k\left(n\right)=\Theta \left(n^{\frac{1}{2}}\right)$. However, the convergence result in [11] (Theorem 1) is an explicit bound, so the condition on k(n) can be relaxed (choosing a smaller k(n)) when we are only interested in the asymptotic behavior.*

#### 3.4.3. Convergence of the Overall Estimation

To complete our analysis on the consistency of the neural estimator, it is required to show that the loss function is properly approximated and it converges to the optimal loss as *n* increase. The following assumptions on the neural network and the densities enable us to show this convergence.

**Assumption** **4.** *For a network $\omega_{\theta }$ parameterized with θ∈Θ, the assumption holds if *Θ* is closed, $\Theta \subseteq \{\theta |∥\theta {∥}_{2}\leq K\}$ for some constant K>0 and $\omega_{\theta }$ is B-Lipschitz, for some constant B>0, regarding θ, for all (x,y,z), i.e.,*

$$\left|\omega_{\theta_{1}}\left(x,y,z\right)-\omega_{\theta_{2}}\left(x,y,z\right)\right|\leq B{∥\theta_{1}-\theta_{2}∥}_{2},\;\forall \theta_{1},\theta_{2}\in \Theta ,\left(x,y,z\right)\in X\times Y\times Z.$$

**Assumption** **5.** *There exist $0<p_{\min}<p_{\max}<\infty$ such that for all x,y,z∈X×Y×Z, the values of p(x,y,z) and p(x|z)p(y,z) are both in the interval $\left[p_{\min},p_{\max}\right]$, and it holds that*

(28) $$\begin{array}{c} \frac{p_{\min}}{p_{\max}+p_{\min}}\geq \tau , \end{array}$$

to guarantee that $\tau \leq \omega^{*}\leq 1-\tau$.

The following theorem concludes the consistency of the end-to-end estimator.

**Theorem** **1.** *Let Assumptions 1, 2, 3, 4, and 5 hold and k(n) and s(n) satisfy *(25)*. Then the CMI estimator ${\hat{I}}_{DV}^{n}\left(X;Y|Z\right)$ (defined in *(21)*), converges strongly to I(X;Y|Z), i.e.,*

(29) $$\begin{array}{c} {\hat{I}}_{DV}^{n}\left(X;Y|Z\right)\overset{a.s.}{\to }I\left(X;Y|Z\right). \end{array}$$

**Proof.**  See Appendix D. ☐

In the next section, we apply our estimator in several synthetic scenarios to verify its capability in estimating CMI and DI.

## 4. Simulation Results

In this section, we experiment with our proposed estimator of CMI and DI in the following auto-regressive model which is widely used in different applications, including wireless communications [42], defining causal notions in econometrics [43], and modeling traffic flow [44], among others:

(30) $$\left[\begin{array}{c} X_{i}\\ Y_{i}\\ Z_{i} \end{array}\right]=A\left[\begin{array}{c} X_{i}\\ Y_{i}\\ Z_{i} \end{array}\right]+B\left[\begin{array}{c} X_{i-1}\\ Y_{i-1}\\ Z_{i-1} \end{array}\right]+\left[\begin{array}{c} N_{i}^{x}\\ N_{i}^{y}\\ N_{i}^{z} \end{array}\right],$$

where *A* and *B* are 3×3 matrices and the rest of variables are *d*-dimensional row vectors. *A* models the instantaneous effect of $X_{i}$, $Y_{i}$, and $Z_{i}$ on each other and its diagonal elements are zero, while *B* models the effect of previous time instance. $N_{i}^{x}$, $N_{i}^{y}$, and $N_{i}^{z}$ (denoted as noise in some contexts) are independent and generated i.i.d. according to zero-mean Gaussian distributions with covariance matrices $\sigma_{x}^{2}I_{d}$, $\sigma_{y}^{2}I_{d}$, and $\sigma_{z}^{2}I_{d}$, respectively (i.e., the dimensions are *d* and components are uncorrelated). Please note that this model fulfills Assumptions 1 and 2 by setting appropriate initial random variables. Although the Gaussian random variables do not range in a compact set and thus, Assumption 3 does not hold, we could use truncated Gaussian distributions. Such adjustment does not significantly change the statistics of the generated dataset since the probability of finding a value far away from the mean is negligible.

In the following section, we test the capability of our estimator in estimating both conditional mutual information (CMI) and directed information (DI). In both cases, *n* samples are generated from the model and the estimations are performed according to Algorithms 1 and 2. Then according to Algorithm 3, the joint and product batches are split randomly in half to construct train and evaluation sets. Then the parameters of the classifier are trained with the train set and the final estimation is computed with the evaluation set (Codes are available at https://github.com/smolavipour/Neural-Estimator-of-Information-non-i.i.d, accessed on 20 May 2021).

To verify the performance of our technique, we also compared it with the approach taken in [4,31] which is as follows. Conditional mutual information can be computed by subtracting two mutual information terms, i.e.,

(31) $$\begin{array}{c} I(X;Y|Z)=I(X;Y,Z)-I(X;Z). \end{array}$$

So instead of estimating the CMI term directly, one can use a neural estimator such as the classifier based estimator in [4] or the MINE estimator [1], and estimate each MI term in (31) to estimate the CMI. In what follows, we refer to this technique as MI-diff since it computes the difference between two MI terms.

### 4.1. Estimating Conditional Mutual Information

In this scenario, we estimate $I(X_{1};Y_{1}|Z_{1})$ when *A* and *B* are chosen to be:

$$A=\left[\begin{array}{ccc} 0 & 1 & 0\\ 0 & 0 & 0\\ 0 & 0 & 0 \end{array}\right],\;B=\left[\begin{array}{ccc} 0 & 1 & 0\\ 0 & 0 & 1\\ 0 & 0 & 0 \end{array}\right].$$

Then from (30), the CMI can be computed as below:

(32) $$\begin{array}{cc} I(X_{1};Y_{1}|Z_{1}) & =h\left(X_{1}|Z_{1}\right)-h\left(X_{1}|Y_{1},Z_{1}\right)\\ \; & =h\left(Y_{0}+Y_{1}+N_{1}^{x}|Z_{1}\right)-h\left(Y_{0}+N_{1}^{x}|Y_{1},Z_{1}\right)\\ \; & =h\left(Y_{0}+Y_{1}+N_{1}^{x}\right)-h\left(Y_{0}+N_{1}^{x}\right)\\ \; & =\frac{d}{2}\log\left(1+\frac{\sigma_{y}^{2}+\sigma_{z}^{2}}{\sigma_{x}^{2}+\sigma_{y}^{2}+\sigma_{z}^{2}}\right). \end{array}$$

**Figure 3:**
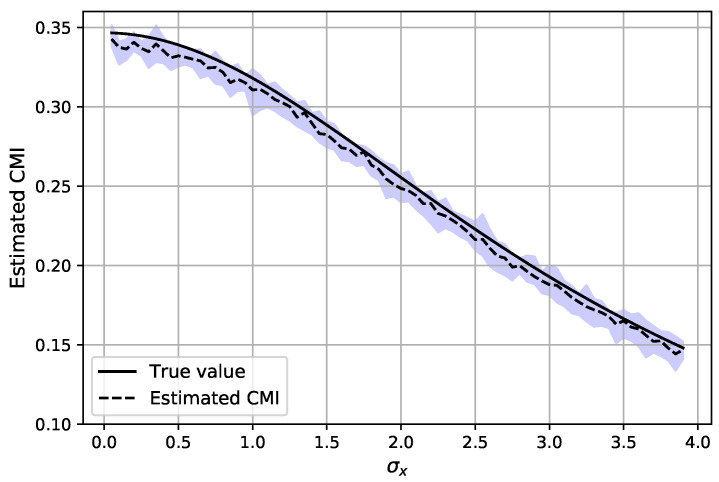
Estimated CMI for AR-1 model in (30) using $n=2\times 10^{4}$ samples with d=1. The shaded region shows the range of the estimated values over the Monte Carlo trials.

**Figure 4:**
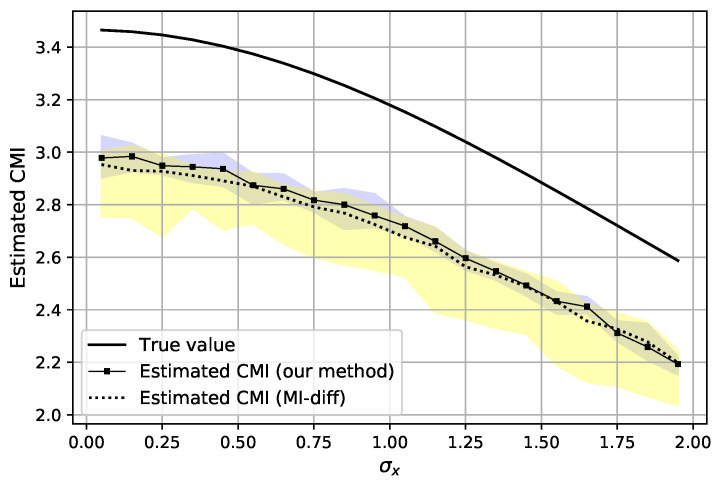
Estimated CMI for AR-1 model in (30) using $n=2\times 10^{4}$ samples with d=10. The shaded region shows the range of the estimated values over the Monte Carlo trials. Blue shades correspond to estimation with our method, yellow shades correspond to estimation with MI-diff approach and the green shade is the overlap of the areas.

Each estimated value is an average of T=20 estimations, where in each round the batches are re-selected while having a fixed dataset. This procedure is repeated for 10 Monte Carlo trials and the data are re-generated for each trial. The hyper-parameters and settings of the experiment are provided in Table 1. In Figure 3, the CMI is estimated (as ${\hat{I}}_{DV}^{n,T}\left(X_{1};Y_{1}|Z_{1}\right)$ in Algorithm 3) with $n=2\times 10^{4}$ samples with dimension d=1 when $\sigma_{y}=2$, $\sigma_{z}=2$ and by varying $\sigma_{x}$. It can be observed that the estimator can properly estimate the CMI while the variance of the estimation is also small. The latter can be inferred from the shaded region, which indicates the range of estimated CMI for a particular $\sigma_{x}$ over all Monte Carlo trials. Next, the experiment is repeated for d=10 and the results are depicted in Figure 4, where we compare our estimation of CMI with the *MI-diff* approach, which is explained in (31) and each MI term is estimated with the classifier-based estimator proposed in [4]. It can be observed that the means of both estimators are similar; nonetheless, estimating the CMI directly is more accurate and has less variation compared to the *MI-diff* approach. Additionally, our method is faster since it computes the information term only once, while in the *MI-diff* approach, two different classifiers are trained to estimate each MI term.

### 4.2. Estimating Directed Information

DI can explain the underlying causal relationship among processes. This notion has wide applications in various areas. For example, consider a social network where the activities of users are monitored (e.g., the messages times as studied in [23]). The DI between these time-series data expresses how the activity of one user can affect the activity of the others. In addition, to such data analytic applications, DI characterizes the capacity of communication channels with feedback and by estimating the capacity, rates and powers of transmission can be adjusted in radio communications (see for example [32]). Now in this experiment, consider a network of three processes X, Y, and Z, such that the time-series data are modeled with (30) with d=1 where

(33) $$\begin{array}{c} A=0,\;B=\left[\begin{array}{ccc} 0 & 0 & 0\\ b_{1} & 0 & 0\\ 0 & b_{2} & 0 \end{array}\right]. \end{array}$$

**Figure 5:**
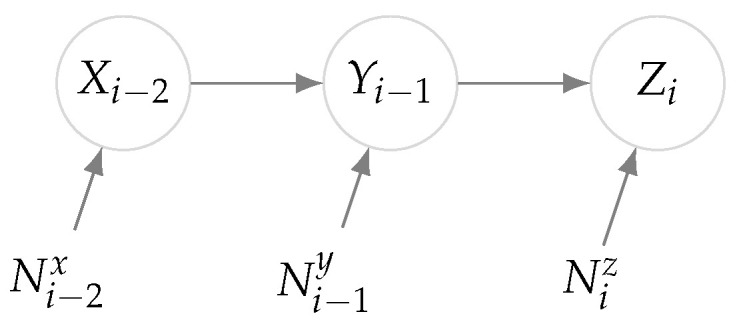
Causal relationship of the processes.

In this model, where the relations are depicted in Figure 5, the process X is affecting Y with a delay and similarly the signal of Y appears on Z in the next time instance while an independent noise is accumulated on both steps. The DIR from X→Y in this network can be computed as follows:

(34) $$\begin{array}{cc} I(X\to Y) & =\lim_{n\to \infty }\frac{1}{n}\sum_{i=1}^{n}I\left(X^{i};Y_{i}|Y^{i-1}\right)\\ \; & =\lim_{n\to \infty }\frac{1}{n}\sum_{i=1}^{n}H\left(Y_{i}|Y^{i-1}\right)-H\left(Y_{i}|X^{i},Y^{i-1}\right)\\ \; & =\lim_{n\to \infty }\frac{1}{n}\sum_{i=1}^{n}H\left(Y_{i}\right)-H\left(Y_{i}|X_{i-1}\right)\\ \; & =\frac{1}{2}\log\left(1+\frac{b_{1}^{2}\sigma_{x}^{2}}{\sigma_{y}^{2}}\right). \end{array}$$

Similarly, for the link Y→Z, we have:

(35) $$\begin{array}{cc} I(Y\to Z) & =\frac{1}{n}\sum_{i=1}^{n}I\left(Y^{i};Z_{i}|Z^{i-1}\right)\\ \; & =\frac{1}{n}\sum_{i=1}^{n}H\left(Z_{i}|Z^{i-1}\right)-H\left(Z_{i}|Y^{i},Z^{i-1}\right)\\ \; & =\frac{1}{n}\sum_{i=1}^{n}H\left(Z_{i}\right)-H\left(Z_{i}|Y_{i-1}\right)\\ \; & =\frac{1}{2}\log\left(1+\frac{b_{1}^{2}\;b_{2}^{2}\;\sigma_{x}^{2}+b_{2}^{2}\;\sigma_{y}^{2}}{\sigma_{z}^{2}}\right). \end{array}$$

Next we can compute the true DIR for the link X→Z as:

(36) $$\begin{array}{cc} I(X\to Z) & =\lim_{n\to \infty }\frac{1}{n}\sum_{i=1}^{n}I\left(X^{i};Z_{i}|Z^{i-1}\right)\\ \; & =\lim_{n\to \infty }\frac{1}{n}\sum_{i=1}^{n}H\left(Z_{i}|Z^{i-1}\right)-H\left(Z_{i}|X^{i},Z^{i-1}\right)\\ \; & =\lim_{n\to \infty }\frac{1}{n}\sum_{i=1}^{n}H\left(Z_{i}\right)-H\left(Z_{i}|X_{i-2}\right)\\ \; & =\frac{1}{2}\log\left(1+\frac{b_{1}^{2}\;b_{2}^{2}\;\sigma_{x}^{2}}{b_{2}^{2}\;\sigma_{y}^{2}+\sigma_{z}^{2}}\right). \end{array}$$

**Figure 6:**
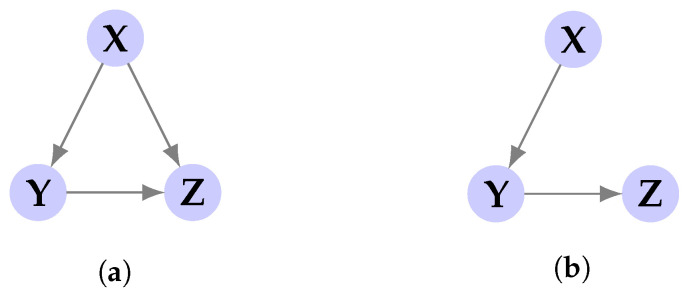
Graphical representation of the causal influences between the processes using pairwise directed information (**a**), and causally conditioned directed information (**b**).

Please note that the DIR corresponding to other links (i.e., the above links in the reverse direction) is zero by similar computations. Suppose we represent the causal relationships with a directed graph, where a link between two nodes exists if the corresponding DIR is non-zero. Then according to (34)–(36), the causal relationships are described with the graph of Figure 6a.

To estimate the DIR, note that the processes are Markov and the *maximum Markov order* ($o_{\max}$) for the set of all processes is $o_{\max}=2$ according to (30) and (33). Hence by (7), we can estimate DIR with the CMI estimator. For instance the DIR for processes (X,Y) can be obtained by:

$${\hat{I}}_{DV}^{n}\left(X\to Y\right):={\hat{I}}_{DV}^{n}\left(X^{3};Y_{3}|Y^{2}\right),$$

where the right hand side is computed similar to (21). We performed the experiment with $n=2\times 10^{5}$ samples of dimension d=1 generated according to the model (30) and (33) with $b_{1}=1$, $b_{2}=2$, $\sigma_{x}=3$, $\sigma_{y}=2$, and $\sigma_{z}=1$, while the settings of the neural network were chosen as in Table 1. The estimated values are stated in Table 2. It can be seen that the bias of the estimator is fairly small while the variance of the estimations is negligible. This is inline with the observations in [11] when estimating CMI for i.i.d. case.

Although I(X→Z)>0, intuitively X is only affecting Z causally through Y, which suggests that I(X→Z∥Y)=0. This event is referred to as *proxy effect* when studying directed information graph (see [45]). In fact the graphical representation of causal relationships can be simplified using the notion of causally conditioned DIR as depicted in Figure 6b. To see this formally, note that from (30) it yields that:

(37) $$\begin{array}{cc} I(X\to Z\;∥\;Y) & =\lim_{n\to \infty }\frac{1}{n}\sum_{i=1}^{n}I\left(X^{i};Z_{i}|Y^{i},Z^{i-1}\right)\\ \; & =\lim_{n\to \infty }\frac{1}{n}\sum_{i=1}^{n}H\left(Z_{i}|Y^{i},Z^{i-1}\right)-H\left(Z_{i}|X^{i},Y^{i},Z^{i-1}\right)\\ \; & =\lim_{n\to \infty }\frac{1}{n}\sum_{i=1}^{n}H\left(Z_{i}|Y_{i-1}\right)-H\left(Z_{i}|Y_{i-1}\right)\\ \; & =0. \end{array}$$

Considering $o_{\max}=2$, the causally conditioned DIR terms can be estimated with our CMI estimator according to (7); for instance,

$$\begin{array}{c} {\hat{I}}_{DV}^{n}\left(X\to Y\;∥\;Z\right):={\hat{I}}_{DV}^{n}\left(X^{3};Y_{3}|Y^{2},Z^{3}\right). \end{array}$$

The estimation results are provided in Table 3 for all the links, where for each link we averaged over T=20 estimations (as in Algorithm 3); then the procedure is repeated for 10 Monte Carlo trials in which we generate a new dataset according to the model.

In this experiment, we did not explore the effect of higher dimensions for data, although one should note that for the causally conditioned DIR estimation, with d=1 the neural network is fed with data of size 9. Nevertheless, the performance of higher dimensions for this estimator with i.i.d. data has been studied in [11] and the challenges of dealing with high dimensions when data has dependency can be considered to be a future direction of this work. Additionally, although the information about $o_{\max}$ may not always be available in practice, it can be approximated by data-driven approaches similar to the method described in [45].

## 5. Conclusions and Future Directions

In this paper, we explored the potentials of a neural estimator for information measures when there exist time dependencies among the samples. We extended the analysis on the convergence of the estimation and provided experimental results to show the performance of the estimator in practice. Furthermore, we compared our estimation method with a similar approach taken in [4,31] (which we denoted as MI-diff), and demonstrations on synthetic scenarios show that the variances of our estimations are smaller. However, the main contribution is the derivation of proofs of convergence when the data are generated from a Markov source. Our estimator is based on a *k*-NN method to re-sample the dataset such that the empirical average over the samples converges to the expectation with certain density. The convergence result derived for the re-sampling technique is stand-alone and can be adopted in other sampling application.

Our proposed estimator can be used potentially in the areas of information theory, communication systems, and machine learning. For instance, the capacity of channels with feedback can be characterized with directed information and estimated with our estimator and can be investigated as a future direction. Furthermore, in machine learning applications where the data has some form of dependency (either spatial of temporal), regularizing the training with information flow requires the estimator of information to capture causality which is considered in our technique. Finally, information measures can be used in modeling and controlling a complex system and the results in this work can provide meaningful measures such as conditional dependence and causal influence.

## Figures and Tables

**Table 1:** Hyper-parameters.

Hidden units	64
Hidden layers	2 (64 × 64)
Activation	ReLU
τ	$10^{-3}$
Optimizer	Adam
Learning rate	$10^{-3}$
Epochs	200

**Table 2:** True and estimated DIR.

	True DIR	Estimation with Our Method (Mean ± Std)
I(X→Y)	0.59	0.57±0.00
I(X→Z)	0.57	0.55±0.00
I(Y→Z)	1.99	1.92±0.01
I(Y→X)	0	0.00±0.00
I(Z→X)	0	0.00±0.00
I(Z→Y)	0	0.00±0.00

**Table 3:** True and estimated DIR.

	True DIR	Estimation with Our Method (Mean ± Std)
I(X→Y∥Z)	0.59	0.57±0.00
I(X→Z∥Y)	0	0.00±0.00
I(Y→Z∥X)	1.42	1.52±0.01
I(Y→X∥Z)	0	0.01±0.00
I(Z→X∥Y)	0	0.01±0.00
I(Z→Y∥X)	0	0.01±0.00

## Data Availability

Not applicable.

## Abbreviations

The following abbreviations are used in this manuscript:

PDF	Probability density function
IID	Independent and identically distributed
MI	Mutual information
CMI	Conditional mutual information
DI	Directed information
DIR	Directed information rate
TE	Transfer entropy
DV	Donsker-Varadhan
NWJ	Nguyen-Wainwright-Jordan
*k*-NN	*k* nearest neighbors
ML	Machine learning
RNN	Recurrent neural network

## Appendix A. Proof of Proposition 1

To show the convergence stated in the Proposition, let us first introduce the following lemma which is a variant of Birkhoff’s ergodic theorem for the case where the samples are not necessarily subsequent.

**Lemma** **A1.**
*Let $U^{n}$ be n observations of a stationary and ergodic Markov process where $U_{i}\in U$ and $U\subseteq R^{d}$. Then if E[g(U)] exists and is finite,*

(A1) $$\begin{array}{c} \frac{1}{|I_{\alpha ,n}|}\sum_{j\in I_{\alpha ,n}}g\left(U_{j}\right)\overset{a.s.}{\to }E\left[g\left(U\right)\right], \end{array}$$

*where $I_{\alpha ,n}$ is defined in Definition 2 and the empirical average is considered to be zero when $|I_{\alpha ,n}|=0$.*

**Proof.** Consider $W_{1},\ldots ,W_{n}$ generated i.i.d. and $W_{i}∼Bernoulli\left(\alpha \right)$. From the definition of $I_{\alpha ,n}$, we can write the summation equivalently as

(A2) $$\begin{array}{c} \sum_{j\in I_{\alpha ,n}}g\left(U_{j}\right)=\sum_{i=1}^{n}W_{i}\;g\left(U_{i}\right). \end{array}$$

Since the $W_{i}$’s are independent of $g(U_{i})$, the pairs $\left(W_{i},g\left(U_{i}\right)\right)$ are also stationary and ergodic Markov, so from Birkhoff’s ergodic theorem,

(A3) $$\begin{array}{c} \frac{1}{n}\sum_{i=1}^{n}W_{i}\;g\left(U_{i}\right)-E\;\left[Wg(U)\right]\overset{a.s.}{\to }0, \end{array}$$

and since $E\;\left[Wg(U)\right]=E\;\left[W\right]E\;\left[g(U)\right]=\alpha E\;\left[g(U)\right]$,

(A4) $$\begin{array}{c} \frac{1}{n}\sum_{i=1}^{n}W_{i}\;g\left(U_{i}\right)\overset{a.s.}{\to }\alpha E\;\left[g(U)\right]. \end{array}$$

On the other hand, from the strong law of large numbers:

(A5) $$\begin{array}{c} \frac{|I_{\alpha ,n}|}{n}=\frac{1}{n}\sum_{i=1}^{n}W_{i}\overset{a.s.}{\to }\alpha . \end{array}$$

From (A4) and (A5), and since the summation in (A5) is bounded,

$$\frac{1}{|I_{\alpha ,n}|}\sum_{j\in I_{\alpha ,n}}g\left(U_{j}\right)\overset{a.s.}{\to }E\;\left[g(U)\right]$$

and the proof is complete. ☐

Using Lemma A1, the proof of Proposition 1 becomes trivial by letting $U_{i}=\left(X_{i},Y_{i},Z_{i}\right)$ since the triple is a sample of a jointly stationary ergodic Markov process. Noting that $|I_{\alpha ,n}\left|=\right|B_{joint}^{\alpha }|$ concludes the proof of the Proposition.

## Appendix B. Proof of Proposition 2

To show the convergence of the empirical average over samples in the product batch, we begin by reviewing convergence results for *k*-NN regression.

**Lemma** **A2** ([39] (Theorem 2-a))**.** *Consider the sequence ${\left\{\left(U_{i},V_{i}\right)\right\}}_{i=1}^{n}$ is stationary and geometrically ϕ-mixing (see Definition 4). If $\frac{k(n)}{n}\to 0$ and $\frac{k(n)}{{\left(\log n\right)}^{2}}\to \infty$, then*

(A6) $$\begin{array}{c} \underset{u}{\sup}\left|m_{n}\left(u\right)-m\left(u\right)\right|\overset{a.s.}{\to }0. \end{array}$$

Now to extend Lemma A2 to the case where the samples are randomly selected for the regression, we show the following lemmas.

**Lemma** **A3.**
*Let ${\left\{\left(X_{i},Y_{i},Z_{i}\right)\right\}}_{i=1}^{n}$ be generated under Assumptions 1–3. If $\frac{k(n)}{n}\to 0$ and $\frac{k(n)}{{\left(\log n\right)}^{2}}\to \infty$, and for any y∈Y, $E_{P_{X|Z}}\;\left[g(X,y,Z)|Z=z\right]$ exists and is finite, then we have that, for all y:*

(A7) $$\begin{array}{c} \underset{z}{\sup}\left|\tilde{g}\left(y,z\right)-E_{P_{X|Z}}\;\left[g(X,y,Z)|Z=z\right]\right|\overset{a.s.}{\to }0, \end{array}$$

*where*

$$\tilde{g}\left(y,z\right):=\frac{1}{k(n)}\sum_{j=1}^{k(n)}\;g\left(X_{r_{j}},y,z\right),$$

*and $r_{j}$ refers to the index of the j-th nearest neighbor of z among ${\left\{Z_{i}\right\}}_{i=1}^{n}$.*

**Proof.** The proof follows directly from Lemma A2 as y is fixed in (A7). ☐

**Lemma** **A4.**
*Let ${\left\{\left(X_{i},Y_{i},Z_{i}\right)\right\}}_{i=1}^{n}$ be generated under Assumptions 1–3. Then, if k(n) and s(n) fulfill the assumptions in (25), and for any y∈Y, $E_{P_{X|Z}}\;\left[g(X,y,Z)|Z=z\right]$ exists and is finite, for all y:*

(A8) $$\begin{array}{c} \underset{z}{\sup}\left|\bar{g}\left(y,z,W^{s(n)}\right)-E_{P_{X|Z}}\;\left[g(X,y,Z)|Z=z\right]\right|\overset{a.s.}{\to }0, \end{array}$$

*where*

(A9) $$\begin{array}{c} \bar{g}\left(y,z,W^{s(n)}\right):=\frac{1}{k(n)}\sum_{l\in A^{\alpha^{\prime },k\left(n\right),n,s(n)}\left(z,Z^{n},W^{s(n)}\right)}\;g\left(X_{l},y,z\right), \end{array}$$

*and $A^{\alpha^{\prime },k\left(n\right),n,s(n)}\left(z,Z^{n},W^{s(n)}\right)$ and $W^{s(n)}$ are defined in Definition 3.*

**Proof.** See Appendix C. ☐

**Lemma** **A5.**
*For the sequence ${\left\{\left(X_{i},Y_{i},Z_{i}\right)\right\}}_{i=1}^{n}$ defined in Lemma A4:*

(A10) $$\begin{array}{c} |\bar{g}\left(Y_{s(n)},Z_{s(n)},W^{s(n)}\right)-E_{P_{X|Z}}\;\left[g\left(X,Y,Z\right)|Y=Y_{s(n)},Z=Z_{s(n)}\right]|\overset{a.s.}{\to }0, \end{array}$$

*where s(n)<n and the convergence occurs according to the random variables $Y_{s(n)},Z_{s(n)},W^{s(n)}$, and the sequence.*

**Proof.** To simplify the notation, we use $\bar{g}\left(y,z\right)$ instead of $\bar{g}\left(y,z,W^{s(n)}\right)$ in this proof. Since Y is compact, for any ϵ>0, there exist M finite balls with radius $\epsilon /L_{g}$ and centers ${\tilde{y}}_{j}$ for j=1,…,M, that cover Y. Then, from the triangle inequality, we have:

(A11) $$\begin{array}{cc} \; & P\;\left(\lim_{n\to \infty }\underset{y,z}{\sup}\left|\bar{g}\left(y,z\right)-E_{P_{X|Z}}\left[g\left(X,y,Z\right)|Z=z\right]\right|\leq 2\epsilon \right)\\ \; & \;\geq P\;\left(\lim_{n\to \infty }\underset{y,z}{\sup}|\Delta_{\left(1\right)}\left(y,z\right)|+|\Delta_{\left(2\right)}\left(y,z\right)|+|\Delta_{\left(3\right)}\left(y,z\right)|\leq 2\epsilon \right), \end{array}$$

where

(A12) $$\begin{array}{cc} \; & \Delta_{\left(1\right)}:=\left(y,z\right)\bar{g}\left(y,z\right)-\bar{g}\left({\tilde{y}}_{j},z\right) \end{array}$$

(A13) $$\begin{array}{cc} \; & \Delta_{\left(2\right)}:=\left(y,z\right)\bar{g}\left({\tilde{y}}_{j},z\right)-E_{P_{X|Z}}\left[g\left(X,{\tilde{y}}_{j},Z\right)|Z=z\right] \end{array}$$

(A14) $$\begin{array}{cc} \; & \Delta_{\left(3\right)}\left(y,z\right):=E_{P_{X|Z}}\left[g\left(X,{\tilde{y}}_{j},Z\right)|Z=z\right]-E_{P_{X|Z}}\left[g\left(X,y,Z\right)|Z=z\right], \end{array}$$

and ${\tilde{y}}_{j}$ is the center of the ball containing *y*. Note that

$$\begin{array}{cc} \; & \lim_{n\to \infty }\underset{y,z}{\sup}\left(|\Delta_{\left(1\right)}\left(y,z\right)\left|+\right|\Delta_{\left(2\right)}\left(y,z\right)\left|+\right|\Delta_{\left(3\right)}\left(y,z\right)|\right) \end{array}$$

(A15) $$\begin{array}{cc} \; & \;\leq \lim_{n\to \infty }\underset{y,z}{\sup}|\Delta_{\left(1\right)}\left(y,z\right)|+\lim_{n\to \infty }\underset{y,z}{\sup}|\Delta_{\left(2\right)}\left(y,z\right)|+\lim_{n\to \infty }\underset{y,z}{\sup}\left|\Delta_{\left(3\right)}\left(y,z\right)\right|\left[2\right] \end{array}$$

(A16) $$\begin{array}{cc} \; & \;\leq 2\epsilon +\lim_{n\to \infty }\underset{y,z}{\sup}\left|\Delta_{\left(2\right)}\left(y,z\right)\right|, \end{array}$$

where (A16) follows from (26) and the radius of the balls being $\epsilon /L_{g}$. Thus (A11) yields:

$$\begin{array}{cc} \; & P\;\left(\lim_{n\to \infty }\underset{y,z}{\sup}\left|\bar{g}\left(y,z\right)-E_{P_{X|Z}}\left[g\left(X,y,Z\right)|Z=z\right]\right|\leq 2\epsilon \right)\\ \; & \;\geq P\;\left(\lim_{n\to \infty }\underset{y,z}{\sup}\left|\Delta_{\left(2\right)}\left(y,z\right)\right|\leq 0\right) \end{array}$$

(A17) $$\begin{array}{cc} \; & \;\geq P\;\left(\lim_{n\to \infty }\max_{{\tilde{y}}_{j}}\underset{z}{\sup}\left|\Delta_{\left(2\right)}\left({\tilde{y}}_{j},z\right)\right|\leq 0\right) \end{array}$$

(A18) $$\begin{array}{cc} \; & \;=P\;\left(\max_{{\tilde{y}}_{j}}\lim_{n\to \infty }\underset{z}{\sup}\left|\Delta_{\left(2\right)}\left({\tilde{y}}_{j},z\right)\right|\leq 0\right) \end{array}$$

(A19) $$\begin{array}{cc} \; & \;\geq 1-\sum_{j=1}^{M}P\;\left(\lim_{n\to \infty }\underset{z}{\sup}\left|\Delta_{\left(2\right)}\left({\tilde{y}}_{j},z\right)\right|>0\right) \end{array}$$

(A20) $$\begin{array}{cc} \; & \;=1, \end{array}$$

where (A17) holds by the definition (A13), (A18) follows since ${\tilde{y}}_{j}$ is independent of *n*, and the last step is due to Lemma A4. Finally since (A20) holds for any ϵ>0, according to [46] (Prop 1.13) it is concluded that:

(A21) $$\begin{array}{cc} \; & P\;\left(\lim_{n\to \infty }\underset{y,z}{\sup}\left|\bar{g}\left(y,z\right)-E_{P_{X|Z}}\left[g\left(X,y,Z\right)|Z=z\right]\right|=0\right)=1. \end{array}$$

Consider now the probability space (Ω,F,P). For any y∈Y and z∈Z, $\bar{g}\left(y,z\right)$ can be expressed equivalently as $\bar{g}\left(y,z;\psi \right):\Omega \to R$. Consider the functions $Y_{s(n)}\left(\psi \right):\Omega \to Y$ and $Z_{s(n)}\left(\psi \right):\Omega \to Z$, then from (A21):

(A22) $$\begin{array}{cc} \; & P\;\left(\psi \in \Omega :\lim_{n\to \infty }\left|\bar{g}\left(Y_{s(n)}\left(\psi \right),Z_{s(n)}\left(\psi \right);\psi \right)-E_{P_{X|Z}}\left[g\left(X,Y,Z\right)|Y=Y_{s(n)}\left(\psi \right),Z=Z_{s(n)}\left(\psi \right)\right]\right|=0\right)\\ \; & \;\geq P\;\left(\psi \in \Omega :\lim_{n\to \infty }\underset{y,z}{\sup}\left|\bar{g}\left(y,z;\psi \right)-E_{P_{X|Z}}\left[g(X,y,Z)|Z=z\right]\right|=0\right)\\ \; & \;=1\;, \end{array}$$

which implies that:

(A23) $$\begin{array}{c} |\bar{g}\left(Y_{s(n)},Z_{s(n)},W^{s(n)}\right)-E_{P_{X|Z}}\;\left[g\left(X,Y,Z\right)|Y=Y_{s(n)},Z=Z_{s(n)}\right]|\overset{a.s.}{\to }0, \end{array}$$

and the proof of Lemma A5 is concluded. ☐

Now that the required tools were introduced, we can continue the proof of Proposition 2. From Definition 3 and (A9), the LHS of (27) can be expressed as below:

(A24) $$\begin{array}{cc} \; & \frac{1}{k\left(n\right)|I_{\alpha^{\prime },s(n)}|}\sum_{\left(X,Y,Z\right)\in B_{prod}^{\alpha^{\prime },s}}g\left(X,Y,Z\right)=\frac{1}{|I_{\alpha^{\prime },s(n)}|}\sum_{i=1}^{s(n)}W_{i}\;\bar{g}\left(Y_{i},Z_{i},W^{s(n)}\right). \end{array}$$

Let us define:

$$\Delta_{i}:=\bar{g}\left(Y_{i},Z_{i},W^{s(n)}\right)-E_{P_{X|Z}}\left[g\left(X,Y,Z\right)|Y=Y_{i},Z=Z_{i}\right],$$

and from Lemma A5, we obtain that:

(A25) $$\begin{array}{c} |\Delta_{s(n)}|\overset{a.s.}{\to }0. \end{array}$$

As a result we can show the following strong convergence:

(A26) $$\begin{array}{cc} P\;\left(\lim_{n\to \infty }\frac{1}{s(n)}\sum_{i=1}^{s(n)}W_{i}\Delta_{i}=0\right) & \geq P\;\left(\lim_{n\to \infty }W_{s(n)}\Delta_{s(n)}=0\right) \end{array}$$

(A27) $$\begin{array}{cc} \; & \geq P\;\left(\lim_{n\to \infty }\left|\Delta_{s(n)}\right|=0\right) \end{array}$$

(A28) $$\begin{array}{cc} \; & =1, \end{array}$$

where (A26) holds since s(n)→∞ by (25) and using Cesáro mean ([47] (Theorem 4.2.3)), (A27) holds since $W_{s(n)}\in \left\{0,1\right\}$, and the equality in the last step follows from (A25). In other words,

(A29) $$\begin{array}{c} \frac{1}{s(n)}\sum_{i=1}^{s(n)}W_{i}\;\bar{g}\left(Y_{i},Z_{i},W^{s(n)}\right)-\frac{1}{s(n)}\sum_{i=1}^{s(n)}W_{i}\;E_{P_{X|Z}}\;\left[g\left(X,Y,Z\right)|Y=Y_{i},Z=Z_{i}\right]\overset{a.s.}{\to }0. \end{array}$$

Next since the sequence ${\left\{\left(W_{i},Y_{i},Z_{i}\right)\right\}}_{i=1}^{s(n)}$ is stationary and ergodic, using Birkhoff’s ergodic theorem we have:

(A30) $$\begin{array}{cc} \; & \;\frac{1}{s(n)}\sum_{i=1}^{s(n)}W_{i}\;E_{P_{X|Z}}\left[g\left(X,Y,Z\right)|Y=Y_{i},Z=Z_{i}\right]\\ \; & \;\overset{a.s.}{\to }E_{P_{W}P_{YZ}}\;\left[W\;E_{P_{X|Z}}\;\left[g(X,Y,Z)|Y,Z\right]\right]. \end{array}$$

As *W* is generated independently

(A31) $$\begin{array}{c} E_{P_{W}P_{YZ}}\;\left[W\;E_{P_{X|Z}}\;\left[g(X,Y,Z)|Y,Z\right]\right]=E\left[W\right]\;E_{\tilde{Q}}\left[g(X,Y,Z)\right]. \end{array}$$

To complete the proof, note that

(A32) $$\begin{array}{c} \frac{|I_{\alpha^{\prime },s(n)}|}{s(n)}\overset{a.s.}{\to }E\left[W\right]. \end{array}$$

Therefore, from (A24) and (A29)–(A32), and $|B_{prod}^{\alpha^{\prime },s}\left|=k\left(n\right)\right|I_{\alpha^{\prime },s(n)}|$ we conclude that:

(A33) $$\begin{array}{c} \frac{1}{|B_{prod}^{\alpha^{\prime },s}|}\sum_{\left(X,Y,Z\right)\in B_{prod}^{\alpha^{\prime },s}}g\left(X,Y,Z\right)\overset{a.s.}{\to }E_{\tilde{Q}}\left[g(X,Y,Z)\right], \end{array}$$

and the proof is complete. ☐

## Appendix C. Proof Lemma A4

According to Definition 3, the index set $I_{\alpha^{\prime },s(n)}$ is determined by the sequence $W^{s(n)}$. Therefore, $A^{\alpha^{\prime },k\left(n\right),n,s(n)}\left(z,Z^{n},W^{s(n)}\right)$ denotes the set of indices of the k(n) nearest neighbors of *z* among $\left\{Z_{i}|i\in I_{\alpha^{\prime },s(n)}^{c}\right\}$, unlike in Lemma A3 where the neighbors can be chosen among the whole sequence ${\left\{Z_{i}\right\}}_{i=1}^{n}$. Hence, the first step is to verify the *ϕ-mixing* condition for the *isolated k-NN* method where some indices are excluded. Intuitively, if ${\left\{X_{i},Y_{i},Z_{i}\right\}}_{i=1}^{n}$ is *ϕ-mixing*, then the sequence ${\left\{\left(X_{i},Y_{i},Z_{i}\right)\right\}}_{i\in I_{\alpha^{\prime },s(n)}^{c}}$ is also *ϕ-mixing* since the random jumps make the asymptotic independence (see Definition 4) happen with a faster rate. Nonetheless, we can show that the sequence ${\left\{\left(X_{i},Y_{i},Z_{i}\right)\right\}}_{i\in I_{\alpha^{\prime },s(n)}^{c}}$ satisfy the mixing condition for Lemma A3 which is expressed in the following.

The basis of the proof for Lemma A2 and thus Lemma A3, is Collomb’s inequality [48] (Theorem 2.2.1) which provides a concentration bound similar to Hoeffding’s inequality for *ϕ-mixing* variables. For instance if U is a ϕ-mixing process where $E\left[U_{i}\right]=0$, $|U_{i}|\leq a_{1}$, $E\left[U_{i}^{2}\right]\leq a_{2}$, and $E\left[\;|U_{i}|\right]\leq a_{3}$, the inequality states that:

(A34) $$\begin{array}{c} P\;\left(|\sum_{i=1}^{n}U_{i}|>\epsilon \right)\leq \exp\left(3\sqrt{e}n\frac{\phi_{t}}{t}-a_{4}\epsilon +6a_{4}^{2}n\left(a_{2}+4a_{1}a_{3}\sum_{i=1}^{t}\phi_{i}\right)\right), \end{array}$$

for some integer t<n and real $a_{4}$ such that $a_{1}a_{4}t\leq 1/4$. In order to show a similar inequality for ${\left\{U_{i}\right\}}_{i\in I_{\alpha^{\prime },s(n)}^{c}}$, we have that:

(A35) $$\begin{array}{cc} P\;\left(|\sum_{i\in I_{\alpha^{\prime },s(n)}^{c}}U_{i}|>\epsilon \right) & =P\;\left(|\sum_{i=1}^{n}U_{i}-\sum_{i=1}^{s(n)}W_{i}U_{i}|>\epsilon \right)\\ \; & \leq P\;\left(|\sum_{i=1}^{n}U_{i}|>\epsilon /2\right)+P\;\left(|\sum_{i=1}^{s(n)}W_{i}\;U_{i}|>\epsilon /2\right), \end{array}$$

where both terms in (A35) are bounded with exponential terms and can be dominated by either of them. Thus, as n→∞ and s(n)→∞ (by assumption (25)) both terms tend to zero and Collomb’s inequality applies to the summation over the sub-sequence of samples remained after the isolation. In other words, the required mixing condition holds for the new sequence ${\left\{\left(X_{i},Y_{i},Z_{i}\right)\right\}}_{i\in I_{\alpha^{\prime },s(n)}^{c}}$ and the result in Lemma A2 can be extended to this Lemma.

Next it remains to verify the conditions of Lemma A2 on k(n). From (25) we have,

$$\frac{k(n)}{|I_{\alpha^{\prime },s(n)}^{c}|}\leq \frac{k(n)}{n-s(n)}=\frac{1}{s(n)(1-\frac{1}{k(n)})}\overset{a.s.}{\to }0,$$

which yields that:

(A36) $$\begin{array}{c} \frac{k(n)}{{\left(\log|I_{\alpha^{\prime },s(n)}^{c}|\right)}^{2}}\geq \frac{k(n)}{{\left(\log n\right)}^{2}}\overset{a.s.}{\to }\infty . \end{array}$$

Therefore, the conditions of Lemma A2 hold and from Lemma A3 it follows that for all y∈Y:

(A37) $$\begin{array}{c} \underset{z}{\sup}\left|\bar{g}\left(y,z,W^{s(n)}\right)-E_{P_{X|Z}}\;\left[g(X,y,Z)|Z=z\right]\right|\overset{a.s.}{\to }0, \end{array}$$

which concludes the proof of the Lemma. ☐

## Appendix D. Proof Theorem 1

Based on the universal functional approximation theory of neural networks [37], ref. [4] (Lemma 4) implies that for any $\epsilon_{0}>0$, there exists $\tilde{\theta }\in \Theta$ such that:

(A38) $$\begin{array}{c} |L\left(\omega_{\tilde{\theta }}\right)-L^{*}|<\frac{\epsilon_{0}}{2}, \end{array}$$

where $L^{*}:=L\left(\omega^{*}\right)$ and L(ω) and $\omega^{*}$ were defined in (15). Moreover, from Propositions 1 and 2, for any θ∈Θ, the empirical loss $L_{emp}\left(\omega_{\theta }\right)$ defined in (18) converges asymptotically to the expected loss $L(\omega_{\theta })$. This is obtained by letting $g\left(x,y,z\right)=\log(\omega_{\theta }\left(x,y,z\right))$ and $g\left(x,y,z\right)=\log(1-\omega_{\theta }\left(x,y,z\right))$ in Propositions 1 and 2, respectively, and noting Remark 2. Thus we have:

(A39) $$\begin{array}{c} L_{emp}\left(\omega_{\theta }\right)\overset{a.s.}{\to }L\left(\omega_{\theta }\right). \end{array}$$

Since $\Theta \subset R^{h}$ and ${∥\theta ∥}_{2}\leq K$, ∀θ∈Θ, Θ can be covered with finite N(Θ,r) number of balls of radius *r*, where N(Θ,r) is bounded [49]:

(A40) $$\begin{array}{c} N\left(\Theta ,r\right)\leq {\left(\frac{2K\sqrt{h}}{r}\right)}^{h}. \end{array}$$

Let $\left\{\theta_{1},\ldots ,\theta_{N(\Theta ,r)}\right\}$ denote the centers of the covering balls. Let $j_{n}$ be the index of the ball that $\hat{\theta }$ belongs to, then from the triangle inequality we have:

(A41) $$\begin{array}{cc} \; & \;|L_{emp}\left(\omega_{\hat{\theta }}\right)-L\left(\omega_{\hat{\theta }}\right)\left|\leq \right|L_{emp}\left(\omega_{\hat{\theta }}\right)-L_{emp}\left(\omega_{\theta_{j_{n}}}\right)\left|+\right|L_{emp}\left(\omega_{\theta_{j_{n}}}\right)-L\left(\omega_{\theta_{j_{n}}}\right)|\\ & \;+|L\left(\omega_{\theta_{j_{n}}}\right)-L\left(\omega_{\hat{\theta }}\right)|\\ \; & \;\leq |L_{emp}\left(\omega_{\theta_{j_{n}}}\right)-L\left(\omega_{\theta_{j_{n}}}\right)|+\frac{2Br}{\tau } \end{array}$$

where the second inequality holds due to the Lipschitz continuity of $\omega_{\theta }$ stated in Assumption 4. From the union bound and for any $\epsilon^{\prime }>0$, we have:

$$\begin{array}{cc} \; & P\;\left(\lim_{n\to \infty }\left|L_{e}mp\left(\omega_{\hat{\theta }}\right)-L\left(\omega_{\hat{\theta }}\right)\right|>\frac{\epsilon^{\prime }}{2}\right) \end{array}$$

(A42) $$\begin{array}{cc} \; & \;\leq N\left(\Theta ,r\right)\;P\;\left(\lim_{n\to \infty }\left|L_{e}mp\left(\omega_{\theta_{j_{n}}}\right)-L\left(\omega_{\theta_{j_{n}}}\right)\right|>\frac{\epsilon^{\prime }}{2}-\frac{2Br}{\tau }\right) \end{array}$$

(A43) $$\begin{array}{cc} \; & \;=0, \end{array}$$

where (A42) holds due to (A41), applying a union bound over all centers $\theta_{j}$, and choosing $r<\frac{\epsilon^{\prime }\tau }{4B}$, and the last step follows by exploiting the strong convergence in (A39). As a result, with probability one:

(A44) $$\begin{array}{cc} \lim_{n\to \infty }L\left(\omega_{\hat{\theta }}\right) & \leq \lim_{n\to \infty }L_{emp}\left(\omega_{\hat{\theta }}\right)+\frac{\epsilon^{\prime }}{2} \end{array}$$

(A45) $$\begin{array}{cc} \; & \leq \lim_{n\to \infty }L_{emp}\left(\omega_{\tilde{\theta }}\right)+\frac{\epsilon^{\prime }}{2} \end{array}$$

(A46) $$\begin{array}{cc} \; & =L\left(\omega_{\tilde{\theta }}\right)+\frac{\epsilon^{\prime }}{2} \end{array}$$

(A47) $$\begin{array}{cc} \; & \leq L^{*}+\epsilon^{\prime }, \end{array}$$

where (A44) is obtained from (A43), and (A45) holds since $\hat{\theta }$ minimizes $L_{emp}\left(\omega_{\theta }\right)$, and (A46) follows from (A39). Finally, the last step is derived using (A38) and choosing $\epsilon_{0}=\epsilon^{\prime }$.

To conclude the proof, note that if Assumption 5 holds, from [4] (Lemma 6) and taking similar steps as in [11] (Lemma 8), it is implied that for any given $\epsilon^{\prime }>0$, with probability one as n→∞:

(A48) $$\begin{array}{c} E_{\tilde{P}}\;\left[|\omega^{*}\left(X,Y,Z\right)-\omega_{\hat{\theta }}\left(X,Y,Z\right)||\hat{\theta }\right]\leq \eta ,\\ E_{\tilde{Q}}\;\left[|\omega^{*}\left(X,Y,Z\right)-\omega_{\hat{\theta }}\left(X,Y,Z\right)||\hat{\theta }\right]\leq \eta , \end{array}$$

where $\eta :=\left(1-\tau \right)p_{\max}\sqrt{2\lambda \epsilon^{\prime }/p_{\min}}$, with λ being the Lebesgue measure corresponding to X×Y×Z. Note that the expectations in (A48) are random variables due to $\hat{\theta }$. Let us define $I_{DV}^{n}\left(X;Y|Z\right)$ as:

(A49) $$\begin{array}{c} I_{DV}^{n}\left(X;Y|Z\right):=E_{\tilde{P}}\;\left[\log\hat{\Gamma }\left(X,Y,Z\right)|\hat{\theta }\right]-\log E_{\tilde{Q}}\;\left[\hat{\Gamma }\left(X,Y,Z\right)|\hat{\theta }\right]. \end{array}$$

Thus by the triangle inequality we have:

(A50) $$\begin{array}{cc} \; & \;|{\hat{I}}_{DV}^{n}\left(X;Y|Z\right)-I\left(X;Y|Z\right)|\\ \; & \;\leq |{\hat{I}}_{DV}^{n}\left(X;Y|Z\right)-I_{DV}^{n}\left(X;Y|Z\right)\left|+\right|I_{DV}^{n}\left(X;Y|Z\right)-I\left(X;Y|Z\right)|. \end{array}$$

where ${\hat{I}}_{DV}^{n}\left(X;Y|Z\right)$ was defined in (21).

To bound the first term, note that by the triangle inequality

(A51) $$\begin{array}{cc} |{\hat{I}}_{DV}^{n}\left(X;Y|Z\right)-I_{DV}^{n}\left(X;Y|Z\right)| & \leq \Delta_{DV}+\Delta_{DV}^{\prime }, \end{array}$$

where

$$\Delta_{DV}:=\left||B_{joint}^{\alpha }{|}^{-1}\sum_{X,Y,Z\in B_{joint}^{\alpha }}\log\hat{\Gamma }\left(X,Y,Z\right)-E_{\tilde{P}}\;\left[\log\hat{\Gamma }\left(X,Y,Z\right)|\hat{\theta }\right]\right|$$

and

$$\Delta_{DV}^{\prime }:=\left|\log|B_{prod}^{\alpha^{\prime },s}{|}^{-1}\sum_{X,Y,Z\in B_{prod}^{\alpha^{\prime },s}}\hat{\Gamma }\left(X,Y,Z\right)-\log E_{\tilde{Q}}\;\left[\hat{\Gamma }\left(X,Y,Z\right)|\hat{\theta }\right]\right|.$$

Since $\hat{\Gamma }\left(\cdot \right)$ is bounded as:

$$\frac{\tau }{1-\tau }\leq \hat{\Gamma }\left(X,Y,Z\right)\leq \frac{1-\tau }{\tau },$$

by the Lipschitz continuity of log(·) it follows that:

(A52) $$\begin{array}{cc} |{\hat{I}}_{DV}^{n}\left(X;Y|Z\right)-I_{DV}^{n}\left(X;Y|Z\right)| & \leq \Delta_{DV}+\Delta_{DV}^{\prime \prime }, \end{array}$$

where

$$\Delta_{DV}^{\prime \prime }:=\frac{1-\tau }{\tau }\left||B_{prod}^{\alpha^{\prime },s}{|}^{-1}\sum_{X,Y,Z\in B_{prod}^{\alpha^{\prime },s}}\hat{\Gamma }\left(X,Y,Z\right)-E_{\tilde{Q}}\;\left[\hat{\Gamma }\left(X,Y,Z\right)|\hat{\theta }\right]\right|.$$

Both $\Delta_{DV}$ and $\Delta_{DV}^{\prime \prime }$ converge strongly to zero from Propositions 1 and 2, respectively, i.e., for any given ϵ>0, we have that:

(A53) $$\begin{array}{c} P\;\left(\lim_{n\to \infty }\Delta_{DV}>\epsilon /4\right)=0,\\ P\;\left(\lim_{n\to \infty }\Delta_{DV}^{\prime \prime }>\epsilon /4\right)=0. \end{array}$$

To bound the second term in (A50), using the triangle inequality it yields that:

(A54) $$\begin{array}{cc} |I_{DV}^{n}\left(X;Y|Z\right)-I\left(X;Y|Z\right)| & \leq |E_{\tilde{P}}\left[\log\hat{\Gamma }\left(X,Y,Z\right)-\log\Gamma \left(X,Y,Z\right)|\hat{\theta }\right]|\\ \;\; & \;+|\log E_{\tilde{Q}}\left[\hat{\Gamma }\left(X,Y,Z\right)|\hat{\theta }\right]-\log E_{\tilde{Q}}\left[\Gamma (X,Y,Z)\right]|. \end{array}$$

Thus from (A48) and the Lipschitz continuity of Γ, $\hat{\Gamma }$, and log(·), it follows that:

(A55) $$\begin{array}{cc} \; & P\;\left(\lim_{n\to \infty }\left|E_{\tilde{P}}\left[\log\hat{\Gamma }\left(X,Y,Z\right)-\log\Gamma \left(X,Y,Z\right)|\hat{\theta }\right]\right|>\frac{\eta }{\tau (1-\tau )}\right)=0,]\\ \; & P\;\left(\lim_{n\to \infty }\left|\log E_{\tilde{Q}}\left[\hat{\Gamma }\left(X,Y,Z\right)|\hat{\theta }\right]-\log E_{\tilde{Q}}\left[\Gamma (X,Y,Z)\right]\right|>\frac{\eta }{\tau^{2}}\right)=0. \end{array}$$

Then combining (A50) and (A52)–(A55), it is concluded that with probability one as n→∞

(A56) $$\begin{array}{cc} |{\hat{I}}_{DV}^{n}\left(X;Y|Z\right)-I\left(X;Y|Z\right)|\; & \leq \Delta_{DV}+\Delta_{DV}^{\prime \prime }+\frac{\eta }{\tau (1-\tau )}+\frac{\eta }{\tau^{2}} \end{array}$$

(A57) $$\begin{array}{cc} \; & \leq \frac{\epsilon }{4}+\frac{\epsilon }{4}+\frac{\epsilon }{2}=\epsilon , \end{array}$$

where the last step holds by choosing $\eta =\tau^{2}\left(1-\tau \right)\frac{\epsilon }{2}$, and $\epsilon^{\prime }$ and $\epsilon_{0}$ accordingly. In other words,

$${\hat{I}}_{DV}^{n}\left(X;Y|Z\right)\overset{a.s.}{\to }I\left(X;Y|Z\right),$$

and the proof of Theorem 1 is completed. ☐
